# Switching From Intravenous to Subcutaneous Immunoglobulin Maintenance Treatment in Chronic Inflammatory Demyelinating Polyradiculoneuropathy

**DOI:** 10.1111/jns.70159

**Published:** 2026-08-08

**Authors:** Yelda Su, Pieter A. van Doorn, Bart C. Jacobs, Krista Kuitwaard

**Affiliations:** ^1^ Department of Neurology Erasmus MC Rotterdam the Netherlands; ^2^ Department of Immunology Erasmus MC Rotterdam the Netherlands

**Keywords:** chronic inflammatory demyelinating polyradiculoneuropathy (CIDP), dose–response, intravenous immunoglobulins (IVIg), subcutaneous immunoglobulins (SCIg), treatment response

## Abstract

**Background and Aims:**

Subcutaneous immunoglobulin (SCIg) is an alternative maintenance therapy to intravenous immunoglobulin (IVIg) in chronic inflammatory demyelinating polyradiculoneuropathy (CIDP). The CIDP guideline considers switching from IVIg to SCIg at an equivalent dosage reasonable; however, the optimal dosing strategy is unknown. This case series examines whether a 1:1 IVIg‐to‐SCIg switch maintains clinical stability.

**Methods:**

IVIg‐dependent, clinically stable CIDP patients who switched from IVIg to SCIg in the Erasmus Medical Center were retrospectively evaluated. Clinical deterioration was defined as a decline in grip strength or muscle strength, or an increase in disability based on minimal clinically important difference criteria within 4 months after the switch.

**Results:**

A total of 10 consecutive CIDP patients on stable IVIg regimens switched to an equivalent (1:1) SCIg dose. One patient remained clinically stable, while nine patients deteriorated. Three of these became clinically stable after increasing the SCIg dose to 1:1.3 or 1:1.5, whereas three patients did not regain stability despite a dose increase to 1:1.3. Clinical stability was maintained in the patient receiving a low dose (0.4 g/kg every 3 weeks), whereas patients receiving higher doses (≥ 1 g/kg every 3 weeks) deteriorated. Five patients switched back to IVIg, while five remained on SCIg throughout follow‐up.

**Interpretation:**

A 1:1 IVIg‐to‐SCIg switch was insufficient to maintain clinical stability in most CIDP patients, including those on higher IVIg maintenance doses. Some but not all patients regained stability after increasing the SCIg dose. Optimal dose adjustment strategies, including whether temporary higher dosing is required, remain to be determined in future studies.

## 1 Introduction

Chronic inflammatory demyelinating polyradiculoneuropathy (CIDP) typically presents with symmetrical sensorimotor deficits, including both proximal and distal weakness in all limbs [1]. CIDP encompasses typical CIDP and CIDP variants, which are generally treated with similar treatment modalities; however, response to treatment varies, with the motor CIDP variant showing a poorer response or even worsening with corticosteroid treatment, and multifocal and distal variants showing a reduced response to intravenous immunoglobulin (IVIg) compared with typical CIDP [2, 3]. IVIg is a well‐established and effective induction and maintenance treatment for CIDP, using either fixed (“one‐size‐fits‐all”) maintenance regimens (1 g/kg every 3 weeks) or individualized dosing schedules adjusted according to clinical response [1, 4]. Subcutaneous immunoglobulin (SCIg) has emerged as a viable alternative for IVIg maintenance therapy in CIDP [5, 6, 7]. The efficacy of SCIg as a maintenance treatment in IVIg‐responsive CIDP patients has been demonstrated in randomized controlled trials [8, 9, 10]. The European Academy of Neurology (EAN)/Peripheral Nerve Society (PNS) guideline for CIDP does not express a preference for either IVIg or SCIg maintenance treatment in CIDP [1]. Advantages of SCIg include increased patient autonomy, elimination of the need for intravenous access, and fewer systemic adverse effects [1, 8, 10]. Moreover, SCIg may reduce long‐term treatment costs and increase autonomy mainly because the patients can shift to home‐based therapy that can be self‐administered also outside of working hours [11, 12].

Despite these benefits, SCIg maintenance treatment is currently only used by a relatively small proportion of patients in The Netherlands. One possible explanation for the limited use of SCIg in clinical practice is the lack of evidence‐based knowledge on how to switch patients from IVIg to SCIg. The EAN/PNS guideline suggests that switching from IVIg to SCIg in an equivalent dosage (1:1) seems reasonable, followed by individualized dose adjustment based on response, noting that some CIDP patients may require higher SCIg doses than their previous IVIg dose [1]. Given differences in bioavailability, a different dosing regimen may be required when switching from IVIg to SCIg [13]. To investigate whether a 1:1 IVIg‐to‐SCIg switch is sufficient to maintain clinical stability, CIDP patients who switched from individualized IVIg maintenance doses to SCIg therapy were evaluated.

## 2 Materials and Methods

All consecutive clinically stable and IVIg‐responsive CIDP patients who were immunoglobulin‐dependent (assessed during routine clinical practice through dose tapering) and switched from IVIg to SCIg maintenance therapy between 2018 and 2025 at the Erasmus University Medical Center were included. Prior to switching all patients received individualized IVIg maintenance therapy, with stable dosing for at least two months. According to our local institutional guideline, treatment is initiated with an induction dose of 2 g/kg IVIg. Patients who have shown objective improvement after 1–2 induction courses but clinically deteriorate thereafter are started on maintenance IVIg treatment at 0.4–1.0 g/kg every 2–4 weeks, depending on the severity of the disease. Dose and interval are subsequently adjusted based on individual clinical response and tolerability, reflecting individualized dosing [14]. All patients fulfilled the European Federation of Neurological Societies (EFNS)/PNS 2010 criteria for the diagnosis of CIDP or the more recent EAN/PNS 2021 criteria [1, 15]. Patients were identified using electronic patient files and by overviews of patients receiving home‐based or hospital day‐care based IVIg or SCIg treatment. This retrospective study used patient chart data. All patients had previously consented to the use of their medical information for research purposes, and ethical approval for the use of these data had been obtained from the institutional ethics committee. The study was conducted in accordance with institutional and national regulations.

A 1:1 switch was defined as switching from IVIg maintenance treatment to an equivalent dosage of SCIg. In clinical practice, patients were switched directly from IVIg to SCIg without overlap or combination of different routes of administration. SCIg was initiated within one to two weeks after the last IVIg infusion. Patient charts were reviewed systematically (YS). All patient cases, in particular the observed efficacy of the 1:1 switch and efficacy of potential dose adjustments, were discussed in a consensus meeting with two neurologists experienced in the diagnosis and treatment of inflammatory neuropathies (PD and KK). Muscle strength was assessed using the Medical Research Council (MRC) sum score (0–60) [16], and hand grip strength with the handheld Martin Vigorimeter (0–160 kPa) [17]. Changes in Inflammatory Neuropathy Cause and Treatment (INCAT) disability scale (0–10) were retrospectively derived when required [18]. Deterioration was based on objective clinical outcome measures, defined by a decline in grip strength, MRC sum score or an increase in INCAT disability scale meeting the minimal clinically important difference (MCID) criteria, and functional changes observed during the first 4 months after the switch [1]. The MCID thresholds used were for grip strength ≥ 8 kPa, MRC sum score ≥ 4, and INCAT disability scale ≥ 1 [1]. The four‐month evaluation window for assessing the efficacy of the 1:1 switch was chosen because steady‐state IgG levels of SCIg are thought to be reached within approximately 8–12 weeks and this four‐month evaluation window has been used in previous studies [19, 20, 21]. Extending the evaluation window beyond 4 months could increase the risk of capturing spontaneous or infection induced fluctuations during the course of the disease.

## 3 Results

A total of 10 CIDP patients (six typical CIDP and four multifocal CIDP) switched from IVIg to an equivalent dose of SCIg (Table 1). The median duration of the maintenance treatment with IVIg was 2.3 years (IQR 1–11.5) and the median dose was 0.6 g/kg/3 weeks (IQR 0.4–0.6). All patients were treated with one of the approved SCIg preparations for CIDP in the Netherlands (Hizentra *n* = 2, HyQvia *n* = 8).

**TABLE 1:** Efficacy and clinical outcomes after switching 1:1 from IVIg to SCIg in CIDP.

Case	CIDP type	Previous IVIg use (years)^1^	Ig dose (g/kg/3 weeks)^2^	Efficacy 1:1 switch^3^	Measures of clinical decline within 4 months^4^, ^5^	Time to first clinical decline after switch (weeks)	Increased IVIg:SCIg ratio within 6 months^6^	Increased SCIg dose (g/kg/3 weeks) within 6 months	Efficacy increased IVIg:SCIg ratio	Rescue Ig (g)^7^	Continued on SCIg
1	Typical	1	0.4	+	n.a.	n.a.	−	−	n.a.	−	+
2	Typical	13	0.6	−	Walking, grip strength, INCAT	16	−	−	n.a.	−	+
3	Typical	1	0.6	−	Endurance, grip strength	12	1.3	0.8	+	−	+
4	Typical	0.5	0.4	−	Weakness	3	1.5	0.6	+	−	+
5	Multifocal	6	0.4	−	Weakness, ADL dependence	3	1.3	0.5	+	−	+
6	Typical	20	1.1	−	Walking, grip strength	2	−	−	n.a.	40 (SCIg), 40 (IVIg)	−
7	Multifocal	11.5	1.0	−	Weakness, grip strength, MRC sum score	12	−	−	n.a.	40 (IVIg)	−
8	Multifocal	3	0.6	−	Weakness, grip strength	12	1.3	0.8	−	−	−
9	Multifocal	1.5	0.6	−	Weakness, grip strength	3	1.3	0.8	−	40 (IVIg)	−
10	Typical	1	0.6	−	Weakness, walking, endurance	3	1.3	0.8	−	3 × 30 (IVIg) 20 (SCIg) 40 (IVIg)	−

Abbreviations: n.a. = not applicable. + = yes, − = no.

^1^ Years of IVIg therapy before switching to SCIg.

^2^ Based on the IVIg dose before switch to SCIg (1:1) and calculated as g/kg every 3 weeks.

^3^ Equal efficacy (IVIg vs. SCIg) in the first four months after the switch from IVIg to SCIg.

^4^ Clinical decline within four months after the switch. For quantifiable measures (grip strength, MRC sum score, INCAT), the MCID cut off values according to the EAN/PNS guidelines were fulfilled.

^5^ Decline in patient 2 fulfilled MCID; however the patient prioritized maintaining her improved autonomy when treated with SCIg.

^6^ Increase in SCIg dosage in the first six months after switch from IVIg to SCIg (dose adjustment factor as compared to the IVIg dose).

^7^ Additional single SCIg or IVIg infusion in grams within the first six months after switch from IVIg to SCIg.

### 3.1 Case 1

A 32‐year‐old female switched from IVIg 30 g every three weeks to SCIg 20 g every two weeks due to poor venous access. After switching, grip strength and MRC sum scores remained stable for at least one year. In addition, during further SCIg treatment functional improvement was observed, as she no longer required regular use of ankle‐foot orthoses and regained the ability to squat. No end‐of‐dose symptoms were reported with either IVIg or SCIg. SCIg‐related adverse effects were transient subcutaneous nodules lasting a few hours at the two infusion sites. At 18 months after switching, she still required SCIg to maintain clinical stability after a prior unsuccessful tapering attempt.

### 3.2 Case 2

A 65‐year‐old female switched from IVIg to SCIg for various reasons such as poor venous access and a desire for increased autonomy due to frequent travel abroad. She switched from IVIg 45 g every three weeks to SCIg 15 g weekly, with initially stable grip strength and MRC sum score. She experienced similar but less severe end‐of‐dose symptoms with SCIg than on IVIg, including slight weakness and increased difficulty climbing stairs before every infusion. Nearly four months after the start of SCIg, neurological examination revealed decreased grip strength, and walking difficulty requiring use of a cane even for shorter distances (INCAT increase of 1 point). However, SCIg was continued without changes to the regimen as the patient prioritized maintaining her improved autonomy. Side effects consisted of local skin irritation and subcutaneous nodules. At 14 months after switching, she still required SCIg treatment.

### 3.3 Case 3

A 70‐year‐old male switched from IVIg to SCIg (both 30 g every two weeks) to increase autonomy while traveling frequently. Shortly after switching to SCIg, he developed sensory end‐of‐dose symptoms. After three months physical endurance and grip strength declined, prompting an increase to 20 g SCIg weekly, which improved grip strength and resolved end‐of‐dose symptoms. As a side effect subcutaneous nodules occurred, which improved when using two infusion sites. Five years after switching, he was still dependent on SCIg.

### 3.4 Case 4

A 52‐year‐old female switched from IVIg 30 g every three weeks to SCIg 10 g weekly due to poor venous access. After three weeks on SCIg, she experienced distal lower limb weakness, resulting in difficulty driving. Consequently, the SCIg dose was adjusted to 30 g every two weeks, restoring muscle strength to baseline and allowing resumption of all usual activities. Side effects included mild local pain and subcutaneous nodules at the two infusion sites, fatigue, and headaches, the latter resembling those experienced during IVIg treatment. She experienced slightly decreased strength at the end of the dosing interval during either treatment. More than two years after switching, she was still dependent on SCIg.

### 3.5 Case 5

A 73‐year‐old male with poor venous access and cardiac comorbidity switched from IVIg 35 g every three weeks to SCIg 20 g every two weeks. After three weeks, he started experiencing more weakness and became dependent on assistance for his daily care. In response, the SCIg dose was adjusted to 30 g every two weeks resulting in partial improvement in muscle strength. As a local reaction, small subcutaneous nodules were reported. At 10 months after switching, he was still in need of SCIg maintenance treatment.

### 3.6 Case 6

Due to cardiac comorbidity and a desire for increased autonomy, a 77‐year‐old male switched from IVIg to SCIg, both 40 g weekly. Two weeks after the switch, a significant reduction in grip strength was measured at the day care unit. The regimen was maintained. Four months after switching, the patient started having increased difficulty walking and received two courses of rescue therapy (40 g of SCIg and 40 g of IVIg). The IVIg rescue medication yielded only a transient clinical benefit, followed by a progressive decline in muscle strength and walking. Reported adverse events during SCIg treatment included subcutaneous nodules, headache during and diarrhea shortly after infusion. After six months of SCIg treatment, he switched back to IVIg, which provided clinical benefit. Two and a half years after re‐initiation of IVIg, he was still IVIg dependent with frequent need for rescue treatment and died due to comorbidity thereafter.

### 3.7 Case 7

Due to poor venous access, a 71‐year‐old male switched from IVIg 60 g every two weeks to SCIg 30 g weekly. While he previously had sensory end‐of‐dose symptoms on IVIg, he had no end‐of‐dose symptoms on SCIg. Three months after the switch, he experienced deterioration of muscle strength, reflected in a significantly reduced MRC sum score and grip strength. An IVIg rescue treatment of 40 g resulted in a recovery of strength, as indicated by improvements in both grip strength and MRC sum score. Following rehabilitation, the patient utilized a walking frame or wheelchair when outside the home; prior to the switch he had already relied on a mobility scooter. Over time, he required multiple IVIg and SCIg rescue treatments. Side effects during SCIg therapy included localized swelling at the infusion site and fatigue. After 24 months of SCIg therapy, IVIg was reintroduced which led to marked improvement. IVIg rescue treatments were required 3 and 18 months later. Almost seven years after the initial SCIg switch, he was still dependent on IVIg maintenance treatment.

### 3.8 Case 8

A 59‐year‐old male switched from IVIg to SCIg both 30 g every two weeks, preferring increased autonomy. Three months thereafter, he experienced increased weakness and reduced grip strength. In response, the SCIg regimen was adjusted to 15 g weekly. By the six‐month follow‐up visit, side effects (localized redness and subcutaneous nodules) had decreased, but grip strength remained reduced. Due to persistent symptoms, the dose was escalated to 20 g weekly. Despite the SCIg increase, significant hand weakness persisted, impairing his ability to use tools at work. After eight months of SCIg therapy, IVIg was re‐initiated. Following three IVIg infusions, he regained his prior functional level. Five years after the initial SCIg switch, he was still IVIg dependent.

### 3.9 Case 9

A 46‐year‐old male switched from IVIg 40 g every two weeks to SCIg 20 g weekly to gain more autonomy. He had no end‐of‐dose symptoms on either treatment. Three weeks after switching, he developed a persistent reduction in muscle and grip strength, but because dexterity seemed to improve, the SCIg dose remained unchanged. After five months of persistent weakness, an IVIg rescue treatment of 40 g led to temporary improvement. During the six months of follow‐up, grip strength slowly worsened. SCIg dose was increased to 25 g weekly, without reported improvement. Adverse effects on SCIg were similar to those on IVIg (fatigue and headache), with the addition of subcutaneous nodules associated with SCIg. Due to deterioration and increased severity of adverse effects, IVIg was reinitiated following a nine‐month period of SCIg treatment. He regained his strength and continued IVIg. Five years after the initial SCIg switch, he was still in need of IVIg therapy.

### 3.10 Case 10

Due to frequent work‐related travel, a 46‐year‐old woman switched from IVIg 30 g every two weeks to SCIg 15 g weekly. No end‐of‐dose symptoms occurred with either treatment. However, after three weeks she developed progressive weakness (with inability to squat, reduced endurance, and difficulty walking) which did not improve after increasing the SCIg dose to 20 g weekly. This prompted three IVIg rescue treatments (30 g each) over two weeks, resulting in clear improvement in strength and function. However, after three months on SCIg maintenance her strength deteriorated again, with limited response to a SCIg rescue treatment of 20 g—she had more difficulty walking and getting up and was unable to work. A subsequent IVIg rescue treatment of 40 g led to temporary improvement of strength and endurance. SCIg was attempted for a total of six months due to patient preference for autonomy. Adverse reactions of SCIg included local irritation and flu‐like symptoms, the latter of which was also experienced after IVIg treatment. After switching back to IVIg due to clinical deterioration on SCIg, her strength improved substantially, with restoration of squatting ability and walking capacity of 6–8 km daily. Four years after switching back to IVIg, IVIg was successfully tapered and discontinued after a prolonged period of stability. After a treatment‐free interval of 6 months she relapsed following infection, with good response to IVIg re‐initiation. Six years after the initial SCIg switch (one year after IVIg re‐initiation), she was still on IVIg.

## 4 Discussion

Switching from IVIg to SCIg at an equivalent (1:1) dosing was not sufficient to maintain clinical stability in most (9 out of 10) patients on prior individualized and stable IVIg maintenance regimens. Clinical stability was regained in some patients after increasing the SCIg dose, while others remained unstable or switched back to IVIg. Clinical deterioration was the main reason for discontinuation of SCIg. Nevertheless, 5 out of 10 patients ultimately continued SCIg treatment throughout follow‐up, thereby avoiding a return to regular IVIg maintenance therapy and retaining the practical advantages associated with SCIg treatment. In our case series, treatment with higher absolute immunoglobulin doses (≥ 1 g/kg every 3 weeks) did not consistently correlate with a successful 1:1 IVIg‐to‐SCIg switch. The only patient who remained stable after a 1:1 switch had been receiving a lower‐than‐standard IVIg maintenance dose (0.4 g/kg/3 weeks), whereas both patients on ≥ 1 g/kg/3 weeks of IVIg deteriorated after a 1:1 switch to SCIg. This suggests that an unsuccessful 1:1 switch cannot be fully explained by an individualized and lower dose than a standard “one‐size‐fits‐all” 1 g/kg/3 weeks dose [1, 18].

An explanation for the lower efficacy of SCIg when switched 1:1 from IVIg may be its lower bioavailability, which has been reported to be approximately 65%–69% of that of IVIg based on area under the curve (AUC) analyses and the increment in serum IgG levels [13]. Another possible explanation for the reduced efficacy of SCIg is differences in IgG exposure patterns over time, with SCIg providing more stable but lower peak concentrations [19]. Because steady‐state IgG levels after SCIg dose adjustments take approximately 8–12 weeks to stabilize [19, 20], it could be hypothesized that a higher SCIg dose may only be needed temporarily. Variability in the time to clinical deterioration after switching and a possible transient requirement for increased SCIg dosages may reflect individual differences in achieving steady‐state and sufficient IgG levels. The possibility that a higher SCIg dose may only be required transiently after switching is supported by the feasibility of dose reductions during the maintenance phase of SCIg therapy [22].

Additional factors may have influenced the observed response. One patient (patient 7) required several rescue treatments during follow‐up in a period with urinary tract infections and hospitalizations for bladder cancer. Nonetheless, his marked improvement after switching back to his previous IVIg regimen suggests that the equivalent SCIg regimen was overall less effective irrespective of the comorbidity. In two patients (8 and 9), clinical deterioration may have been associated with a delay in dose increase after onset of clinical decline. No distinct association was observed between end‐of‐dose symptoms and objective clinical deterioration. Some patients who continued SCIg reported mild end‐of‐dose symptoms while being clinically stable, whereas others with clinical deterioration did not report end‐of‐dose symptoms. The total duration on IVIg treatment did not affect the success of switching from IVIg to SCIg at a 1:1 ratio. We did not observe clear differences between the effects of the two SCIg products used in our patients. SCIg was discontinued in most patients with multifocal CIDP, suggesting it may be less effective, which may reflect previously reported variability in IVIg responsiveness across CIDP subtypes [2, 3].

These observations are consistent with previous reports showing variability in response when switching from IVIg to SCIg. The PATH trial found that a higher dose of SCIg (0.4 g/kg/week; IVIg‐to‐SCIg ratio 1:1.2) led to fewer relapses than a lower dose SCIg (0.2 g/kg/week; IVIg‐to‐SCIg ratio 1:0.6) [8]. The open‐label extension phase of this study revealed increased relapse rates with the lower dose, with most patients improving upon returning to the higher dose [23]. These results suggest that some patients require a higher SCIg dose than the IVIg dose [8, 23]. Two placebo‐controlled trials showed efficacy of SCIg over placebo in patients previously treated with a similar dose of IVIg [9, 10]. A recent observational study showed that 31% of CIDP and 40% of MMN patients did not maintain clinical stability with a 1:1 switch from IVIg to SCIg in routine care, requiring dose increases in one‐third (median ratios: 1:1.2 and 1:1.25). The lower deterioration rates in that study might be due to the different definition of equivalence (1:1.1 instead of 1:1) and the different population since about a quarter of patients had Sjögren syndrome and received concomitant immunomodulatory therapy [24]. In primary immunodeficiencies (PID), switching from IVIg to SCIg required higher mean doses (dose adjustment ratios of approximately 1:1.37 and 1:1.53) to achieve non‐inferior serum IgG exposure (AUC) [19]. Although derived from PID populations, these findings may also be relevant to CIDP, as differences in IgG pharmacokinetics are primarily determined by the route of administration rather than the underlying disease [13]. To date, no randomized trials directly comparing IVIg and SCIg head‐to‐head have been reported.

Beyond pharmacologic and clinical considerations, individual preferences may guide treatment decisions. Patients preferred SCIg over IVIg, [8, 9, 10, 23, 24] citing increased autonomy, reduced treatment time, fewer side effects, preferred administration frequency, and less end‐of‐dose symptoms [10, 23]. In our study, some patients reported more than one reason for switching to SCIg, including poor venous access (*n* = 5), desire for increased autonomy (*n* = 5), and cardiac comorbidity (*n* = 2).

The strength of this study is the inclusion of consecutive, real‐world CIDP patients on stable individualized IVIg maintenance therapy. The limitations include the small sample size, retrospective nature, single‐center design, and use of different IVIg and SCIg regimens and preparations. We cannot exclude that a 1:1 switch would have been more effective if all patients in our study were treated with standard (1 g/kg every 3 weeks) dosages before switching which might have enabled them to reach a yet unknown IgG threshold to maintain clinical stability. Furthermore, in cases with early deterioration followed by prompt dose adjustment (patients 4 and 5), it cannot be excluded that clinical stability might have been regained under the initial 1:1 regimen if the initial regimen had been maintained until steady‐state IgG levels were reached. Conversely, in some patients who deteriorated after a 1:1 switch SCIg dose increase was either delayed or not attempted (patients 6 and 7), which may have affected the likelihood of regaining stability. Finally, we did not apply standardized tapering attempts during SCIg maintenance treatment, so it remains unclear whether dose increase was only temporarily required during or shortly after the switch from IVIg to SCIg.

To our knowledge, this is one of the first studies exploring the effect of switching from individualized IVIg maintenance treatment to SCIg in patients with CIDP. Although the overall efficacy of SCIg maintenance treatment has been established in randomized controlled trials, our findings suggest that CIDP patients may require higher SCIg maintenance dosages when switching from IVIg to SCIg. Prospective studies are needed to determine the optimal dose adjustment required when switching from IVIg to SCIg.

## Conflicts of Interest

YS has no conflicts of interest to declare. PD received research grants from the Prinses Beatrix Spierfonds and The Netherlands Organisation for Health Research and Development. Consulting fees from Annexon, Argenx, Hansa Biopharma, Octapharma, Sanofi and Takeda; and speaker fees from Argenx, Grifols and Octapharma. All were paid to the institution. BJ received research grants from the Prinses Beatrix Spierfonds, Netherlands Organization for Health Research and Development, GBS‐CIDP Foundation International, Sanquin, Annexon, Hansa Biopharma, Roche, Grifols, Octapharma and Argenx. All were paid to the institution. KK received research grants from For Wis(h)dom Foundation, The Albert Schweitzer hospital and Takeda, consulting fees from Takeda and speaker fees from Octapharma, Grifols and Novartis. All were paid to the institution.

## Data Availability

The data that support the findings of this study are available on request from the corresponding author. The data are not publicly available due to privacy or ethical restrictions.
